# Microbiome of Unilateral Chronic Rhinosinusitis: A Controlled Paired Analysis

**DOI:** 10.3390/ijerph18189878

**Published:** 2021-09-19

**Authors:** Sang Chul Park, Il-Ho Park, Joong Seob Lee, Sung Min Park, Sung Hun Kang, Seok-Min Hong, Soo-Hwan Byun, Yong Gi Jung, Seok Jin Hong

**Affiliations:** 1 Department of Otorhinolaryngology-Head and Neck Surgery, Hallym University College of Medicine, Kangnam Sacred Heart Hospital, Seoul 07441, Korea; newliebe@hanmail.net; 2 Department of Otorhinolaryngology-Head and Neck Surgery, Guro Hospital, Korea University College of Medicine, Seoul 08308, Korea; parkil5@korea.ac.kr; 3 Medical Device Usability Test Center, Guro Hospital, Korea University College of Medicine, Seoul 08308, Korea; 4 Department of Otorhinolaryngology-Head and Neck Surgery, Hallym University College of Medicine, Hallym Sacred Heart Hospital, Anyang 14068, Korea; apniosio@hallym.or.kr; 5 Department of Otorhinolaryngology-Head and Neck Surgery, Hallym University College of Medicine, Dongtan Sacred Heart Hospital, Hwaseong 18450, Korea; hahajane@naver.com (S.M.P.); thecell@hallym.or.kr (S.-M.H.); 6 Department of Biomedical Sciences, College of Medicine, Hallym University, Chuncheon 24252, Korea; malice23@nate.com; 7 Department of Oral & Maxillofacial Surgery, Dentistry, Hallym University College of Medicine, Hallym Sacred Heart Hospital, Anyang 14068, Korea; purheit@daum.net; 8 Department of Otorhinolaryngology-Head and Neck Surgery, Samsung Medical Center, Sungkyunkwan University School of Medicine, Seoul 06351, Korea

**Keywords:** microbiome, 16S rRNA sequencing, rhinosinusitis, unilateral sinusitis

## Abstract

The sinonasal microbiota in human upper airway may play an important role in chronic rhinosinusitis (CRS). Thus, this study aimed to investigate the human upper airway microbiome in patients with unilateral CRS, and compare the sinonasal microbiome of the unilateral diseased site with that of a contralateral healthy site. Thirty samples, 15 each from the diseased and healthy sites, were collected from the middle meatus and/or anterior ethmoid region of 15 patients with unilateral CRS during endoscopic sinus surgery. DNA extraction and bacterial microbiome analysis via 16S rRNA gene sequencing were then performed. *Corynebacterium* showed the highest relative abundance, followed by *Staphylococcus* in samples from both the diseased and healthy sites. Further, the relative abundances of *Staphylococcus* and *Pseudomonas* were significantly lower in samples from diseased sites than in those from healthy sites. Conversely, anaerobes, including *Fusobacterium*, *Bacteroides*, and *Propionibacterium*, were abundantly present in samples from both sites, more so in samples from diseased sites. However, the sites showed no significant difference with respect to richness or diversity (*p* > 0.05). Our results indicate that CRS might be a polymicrobial infection, and also suggest that *Corynebacterium* and *Staphylococcus* may exist as commensals on the sinus mucosal surface in the upper respiratory tract.

## 1. Introduction

The human body surface is colonized by several microorganisms, which constitute the microbiota that plays a crucial role in host immunity [1,2]. The introduction of culture-independent bacterial DNA sequencing techniques has enabled the identification of various microbiomes [3], and many studies have suggested the microbiota to play a role in health and human diseases [2,4,5,6,7]. Commensal bacteria are key components in the development of mucosal barrier function and play a key role in innate and adaptive immune responses [2,8,9]. They also function in suppressing the establishment of pathogens [7,10]. Microbiota dysbiosis may be associated with various diseases including asthma [11], allergic rhinitis [12], atopic dermatitis [13], cardiovascular diseases [14], obesity [15], diabetes [16], and neurodegenerative diseases [17]. Moreover, besides serving as a source of immune perturbation, breaching the mucosal barrier during pathological conditions, such as atherosclerosis, type 2 diabetes, non-alcoholic fatty liver disease, obesity, and inflammatory bowel disease, can also induce commensal bacteria to become a chronic inflammatory stimulus to adjacent tissues [4,6,18]. Thus, abnormalities in microbiota and the resulting effects on the immune system deserve attention.

Chronic rhinosinusitis (CRS), which can persist for at least 12 weeks, is a chronic inflammation of the sinonasal mucosa characterized by nasal obstruction, discharge, facial pain, and a decrease in the sense of smell [19]. Although it is considered to be an inflammation-related disease, rather than an infection, commensal resident microbes and pathogens play a crucial role in the initiation and progression of mucosal inflammation [20,21,22]. Further, emerging evidence has improved our understanding of the relationship between CRS and the microbiome of the sinuses and nasal cavities [20,21,23,24,25,26].

Previous studies on the microbiome in CRS have mostly focused on the comparison of the microbial composition of patients with CRS (CRS with and without polyps) with that of healthy subjects [27,28,29,30,31,32]. In addition, some studies have investigated patients with CRS with clinical variables, including asthma, cystic fibrosis, geographical locations, and the relationship between the heterogeneity of CRS and microbiome dysbiosis [27,33,34]. However, the results of these studies are controversial. For example, while some studies showed a decrease in microbiota diversity in patients with CRS compared with that in controls (*p* < 0.05, 29 controls and 47 patients of CRS with asthma [27]; *p* < 0.05, 3 controls and 8 CRS patients [28]), others suggested either an increase in microbiota diversity in patients with CRS (no mention of *p*-value, 12 controls and 30 CRS patients [29]; *p* < 0.05, 6 controls and 38 CRS patients [30]) or no difference between patients and controls (5 controls and 15 patients of CRS [31]; 26 controls and 56 patients of CRS [32]). These conflicting results can be explained by methodological and population differences, different DNA extraction protocols, and the various confounding factors that correspond to different individuals, including age, severity, and phenotype of diseases, genetic background of patients, and antibiotic use around the time of sampling [21]. Patients with other diseases, which may affect microbiota, including allergies, may have different residential microbiota, despite being administered the same antibiotics at the time of sampling, given that the accumulated antibiotics from childhood in different individuals could be different [21,35]. Moreover, populations of individuals respond differently to the microbiota present, and the microbial community may be individualized [36]. Furthermore, the nasal microbiota could be altered owing to surgery, by several factors including alteration of mucociliary clearance and airflow of sinus [37,38,39].

This implies that it is necessary to investigate the microbiota of a diseased site relative to that of a non-diseased (healthy) site in patients with unilateral sinusitis. Therefore, the objective of this study was to examine the upper airway microbiome in human patients with CRS and compare the sinonasal microbiome of the unilateral diseased site with that of a contralateral healthy (control) site.

## 2. Materials and Methods

### 2.1. Study Subjects

Between May 2017 and September 2019, a total of 30 samples were collected from 15 adult patients with unilateral CRS (aged 18–70 years), who visited the Department of Otorhinolaryngology—Head and Neck Surgery. According to the European position paper on rhinosinusitis and nasal polyps (EPOS) 2020 criteria, a CRS diagnosis was made. The diagnosis was confirmed if the nasal symptoms lasted more than 12 weeks, nasal polyps or purulent discharge were present on endoscopic examination, or mucosal inflammation was observed in the sinuses or ostiomeatal unit on computed tomography (CT) [19]. All patients had undergone endoscopic sinus surgery (ESS); 15 samples were obtained from unilateral diseased sinus and another 15 (controls) were obtained from the contralateral healthy sinus that was clean on CT scan and did not show purulent discharges on endoscopy. The exclusion criteria included pregnancy, immunocompromised status, age < 18 years, and other sinonasal diseases, such as acute rhinosinusitis, fungal sinusitis, and tumors. Patients had not taken oral steroids, non-steroidal anti-inflammatory drugs, antihistamines, or antibiotics for at least four weeks prior to the surgery.

Clinical data, including demographic information, such as age and gender, Lund–Mackay CT (LM CT) scores, past medical history, atopy, and asthma, were recorded. The diagnosis of allergy was based on the serum allergen-specific immunoglobulin E (IgE) tests, such as the multiple allergen simultaneous test (MAST) or ImmunoCAP.

All patients provided written informed consent, and all the protocols were approved by the Institutional Review Board of Hallym University College of Medicine (IRB # 2016-524-I). Portions of data on some subjects in this study have been partially presented in a previous study [26]; however, the case number of the study was newly assigned, and the aim and results are different.

### 2.2. Sample Collection

**Figure 1:**
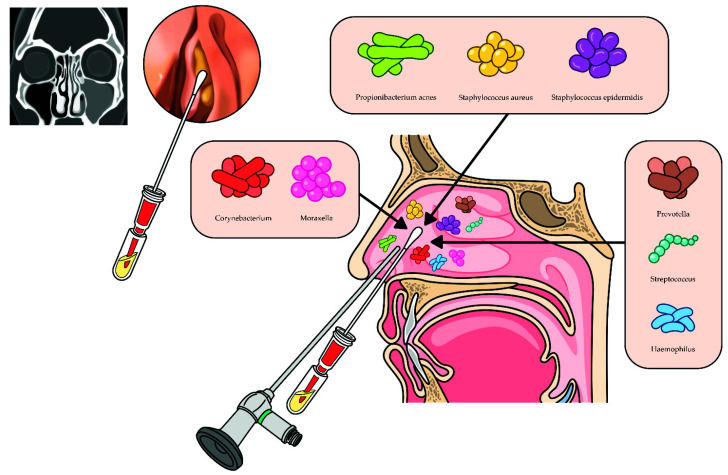
The study design with sampling method.

Samples were collected during ESS under general anesthesia before the administration of topical mucosal vasoconstrictors, including 1:100,000 epinephrine and anesthetics. We used a nasal speculum and endoscope and collected the samples carefully to avoid contamination from the skin around the anterior nostril and nasal vestibule. Pairs of endoscopically guided swab samples were taken from the middle meatus and/or anterior ethmoid region using sterile swabs (Quick Swab, 3M Microbiology, St. Paul, MN, USA), not touching the anterior nostril and nasal vestibule (Figure 1). If it was determined that the specimen was contaminated, the specimen was discarded, and we collected the sample again. After collection, the samples were immediately placed in ice and frozen at −80 °C.

### 2.3. DNA Extraction and Metagenomic Analysis

Total DNA was extracted using the FastDNA SPIN Kit for Soil (MP Biomedicals, USA), following the manufacturer’s instruction. PCR amplification was performed using fusion primers targeting the V3‒V4 regions of the 16S rRNA gene with the extracted DNA. For bacterial amplification, fusion primers were 341F (5′- AATGATACGGCGACCACCGAGATCTACAC-XXXXXXXXTCGTCGGCAGCGTC-AGATGTGTATAAGAGACAG-CCTACGGGNGGCWGCAG-3′; underlined sequence indicates the target region primer) and 805R (5′-CAAGCAGAAGACGGCATACGAGAT-XXXXXXXXGTCTCGTGGGCTCGG-AGATGTGTATAAGAGACAG-GATACHVGGGTATCTAATCC-3′). The fusion primers were constructed in the following order: P5 (P7) graft binding, i5 (i7) index, Nextera consensus, sequencing adaptor, and target region sequence. We purified the amplified products using CleanPCR (CleanNA, Waddinxveen, The Netherlands), pooled together equal concentrations of purified products, and eliminated short fragments (non-target products) using CleanPCR (CleanNA, Waddinxveen, The Netherlands), followed by assessment of the quality and product size on a Bioanalyser 2100 (Agilent, Palo Alto, CA, USA) using a DNA 7500 chip. Next, mixed amplicons were pooled, after which sequencing was performed at ChunLab, Inc. (Seoul, Korea) using an Illumina MiSeq Sequencing system (Illumina, San Diego, CA, USA), following the manufacturer’s instructions [40].

### 2.4. Pyrosequencing Data Analysis

The processing of raw reads began with a quality check and the filtering of low-quality reads (average score < 25) using Trimmomatic v0.32 [41]. After passing the quality check, paired-end sequence data were merged together using the fastq_mergepairs command of VSEARCH v2.13.4, set to default parameters [42]. Further, primers were trimmed using the alignment algorithm proposed by Myers and Miller at a similarity cut-off of 0.8 [43]. Non-specific amplicons that did not encode 16S rRNA were detected using the nhmmer 4 tool in the HMMER software package v3.2.1 with hmm profiles [44]. Unique reads were extracted, and redundant reads were clustered with the unique reads using the derep_fulllength command of VSEARCH [42].

### 2.5. Bioinformatics Analysis

**Figure 2:**
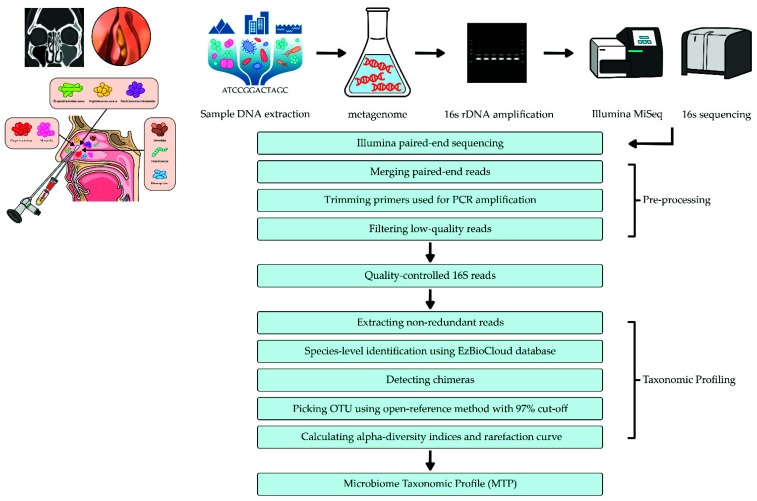
The flowchart on unilateral chronic rhinosinusitis and microbiome phlogistic pathways.

Sequences were merged and checked for quality control. Thereafter, operational taxonomic units (OTUs) were assigned, usually at the genus level, using pre-curated 16S sequence databases. Relative abundance (abundance of an OTU as a percentage of all sequences in a sample) and richness (number of OTUs identified in a sample) were established for further downstream analysis [45]. Additionally, the EzBioCloud database (http://www.ezbiocloud.net/, accessed on 8 August 2021) was used for taxonomic assignment using the usearch_global command of VSEARCH [40,42], while pairwise alignment was employed to calculate similarity [40]. Uchime and the non-chimeric 16S rRNA database from EzBioCloud were used to detect chimera in reads that contained a < 97% best hit similarity rate. Sequence data were then clustered using CD-Hit7 and UCLUST8, and then alpha diversity analysis was conducted (Figure 2).

### 2.6. Statistical Analysis

All statistical analyses were performed using R software version 3.1.2. Comparisons between groups were performed using Wilcoxon signed-rank tests. Further, correlations between two variables were determined by Pearson and Spearman rank correlation tests.

## 3. Results

### 3.1. Sequence Read Counts and Taxonomic Assignments

**Figure 3:**
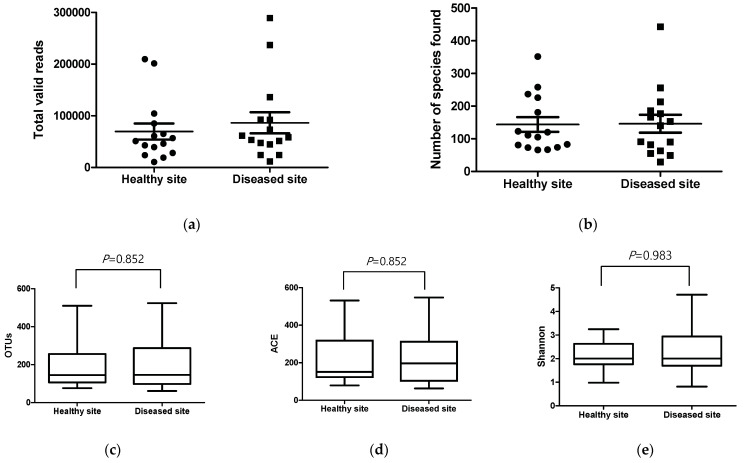
Sequence read counts in samples of healthy site and diseased site. (**a**) Total valid reads per sample in healthy site and diseased site. (**b**) Number of species found per sample in healthy site and diseased site. Comparison of bacterial species richness and alpha diversity in samples of healthy site and diseased site. Richness represented by (**c**) operational taxonomic units (OTU), (**d**) abundance-based coverage estimators (ACE), and alpha diversity examined by (**e**) Shannon index.

We compared swab samples collected during ESS from 15 patients, aged 17–67 years. The demographic and clinical characteristics of the patients are shown in Table 1. The total number of reads was counted, after which the data were prefiltered and passed through the quality check process to determine valid reads (Supplementary Table S1). Thus, an average of 81,640 and 96,572 bacterial 16S rRNA-encoding gene sequence reads were observed in samples from the healthy and diseased sites, respectively. Reads with low-quality amplicons, non-target amplicons, and chimeric amplicons were removed. Further, the rates of observation of valid reads at the healthy and diseased sites, considering the total number of reads at both sites, varied in the ranges 21.0–96.1 and 37.9–96.5, respectively. The mean sequence length, after sequence processing per sample, ranged from 409 to 425 bases. Furthermore, there was no statistically significant difference in the number of valid reads between the healthy and diseased sites (Figure 3a). These findings indicate that both sites have similar bacterial loads. After the valid reads were assigned against reference databases at the species level, the number of reads identified at this level, obtained per sample, varied in the ranges 10,402–208,560 and 11,606–289,190 at healthy and diseased sites, respectively. The taxonomic coverage of the database ranged from 80.8 to 99.9 at both sites, and there was no statistically significant difference between the number of species identified at the healthy and diseased sites (Figure 3b).

### 3.2. Comparison of Richness and Alpha Diversity Indices

Richness is defined as the number of unique species per sample, identified using a reference database. The number of OTUs in samples from healthy and diseased sites varied in the ranges 76–373 (median: 145) and 61–370 (median: 146), respectively. However, these different numbers of OTUs in samples from the two sites showed no statistical significance (*p* = 0.852) (Figure 3c). Additionally, other species richness indices, such as abundance-based coverage estimators (*p* = 0.694) (Figure 3d) and a Jackknife estimator (*p* = 0.568), did not show any statistically significant difference between the two sampling sites (Supplementary Figure S1a). Alpha diversity, which refers to intra-community diversity, was measured based on the Shannon index, Simpson index, and phylogenetic diversity. Based on these indices, there was no statistically significant difference between the two sites (Figure 3e), (Supplementary Figure S1b,c). Beta diversity, which refers to a comparison of diversity between different groups, was evaluated by principal coordinates analysis (PCoA) plots. There was no definite clear separation between the two sites (Supplementary Figure S1d).

### 3.3. Average Composition of Microbiota at Phylum, Genus, and Species Levels

**Figure 4:**
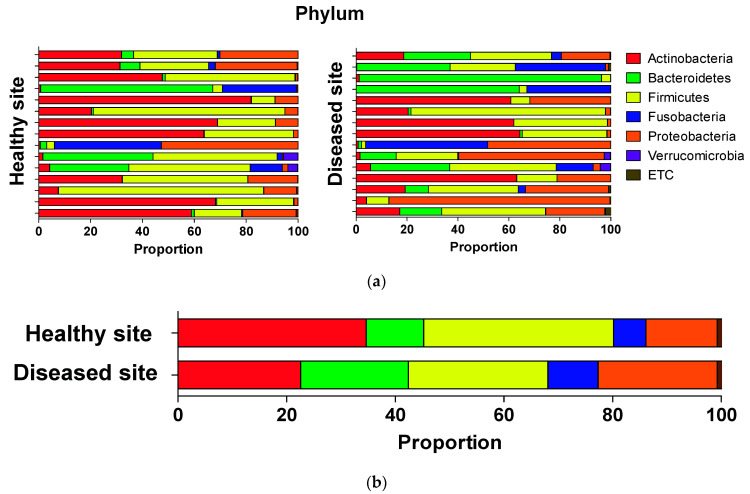
(**a**) Comparison of microbiota per sample between healthy site and diseased site at phylum level. (**b**) Stacked bars show average taxonomic composition of selected communities of bacterial phylum level.

In this study, we also examined the differences in microbiota composition between the diseased and contralateral healthy sites. At the phylum level, five bacterial phyla, *Firmicutes, Actinobacteria, Proteobacteria, Bacteroidetes*, and *Fusobacteria*, showed dominance at both sites (Figure 4a). Further, diseased sites showed slightly lower *Actinobacteria* and *Firmicutes* compositions than healthy sites, which showed slightly higher compositions of *Proteobacteria, Bacteroidetes*, and *Fusobacteria*. However, the differences were not statistically significant (Figure 4b).

**Figure 5:**
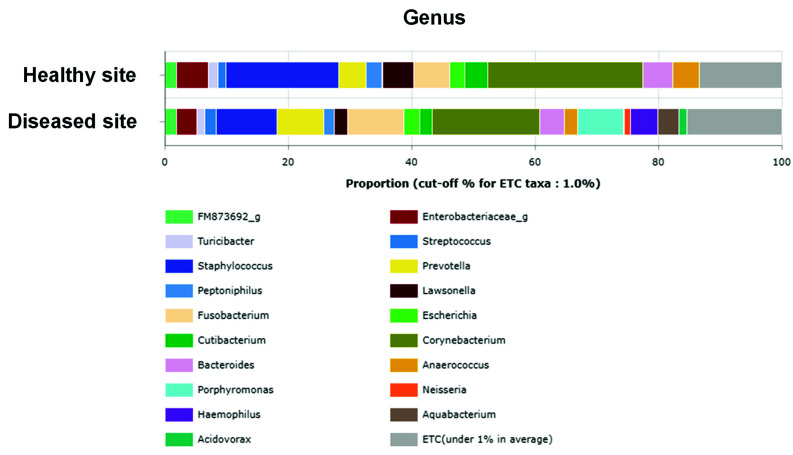
Comparison of microbiota between healthy site (upper bar) and diseased site (lower bar) at genus level. Stacked bars show average taxonomic composition of selected communities of bacterial genus level.

At the genus level, *Corynebacterium* was identified as the most abundant genus in samples from both sinuses (25.11 and 17.48% at healthy and diseased sites, respectively), followed by *Staphylococcus* (18.32%), *Fusobacteria* (5.89%), *Enterobacteriaceae* (5.24%), *Lawsonella* (5.08%), *Bacteroides* (4.89%), *Prevotella* (4.47%), and *Anaerococcus* (4.27%) at healthy sites and *Staphylococcus* (9.79%), *Fusobacterium* (9.08%), *Prevotella* (7.59%), *Porphyromonas* (7.49%), *Haemophilus* (4.50%), and *Bacteroides* (4.06%) at the diseased sites (Figure 5). Further, at the species level, *Corynebacterium spp.*, such as *Corynebacterium accolens* (16.28 and 11.44% at healthy and diseased sites, respectively) and *Corynebacterium tuberculostearicum* (8.39 and 5.07% at healthy and diseased sites, respectively), were prevalent at both sites, while *Staphylococcus aureus* was also prevalent at both sites, showing a tendency to decrease at the diseased sites compared with the control sites (18.39 and 9.55% at healthy and diseased sites, respectively) (Supplementary Figure S2).

### 3.4. Comparison of Relative Abundance at Genus and Species Levels

**Figure 6:**
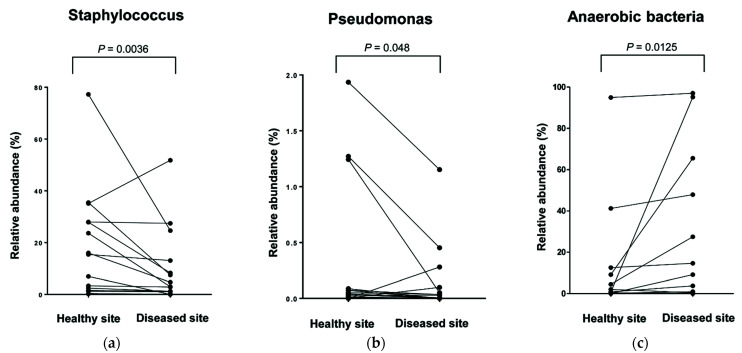
Composition of representative microbiota at genus level of (**a**) Staphylococcus, (**b**) Pseudomonas, and (**c**) anaerobes. Paired line plots show relative abundance of selected communities in comparison between healthy site and diseased site.

Next, we compared the microbiota compositions of the samples collected from the healthy and unilateral diseased sites. At the genus level, the relative abundances of *Staphylococcus, Anaerococcus, Acinetobacter, Halomonas,* and *Pseudomonas* significantly decreased at the diseased sites more so than at the healthy sites (Figure 6a,b). Conversely, the relative abundances of anaerobes, including *Prevotella, Porphyromonas, Fusobacterium,* and *Propionibacterium,* showed a significant increase at the diseased sites (Figure 6c). However, the relative abundances of other microbiota did not show any statistically significant difference between the two sites.

At the species level, the relative abundances of *Staphylococcus aureus, Anaerococcus octavius, Corynebacterium tuberculostearicum,* and *Halomonas stevensii* significantly decreased more so at the diseased site than at the healthy site. Notably, the relative abundances of anaerobes belonging to the anaerobic genus, including *Prevotella, Porphyromonas, Fusobacterium,* and *Propionibacterium*, showed a significant increase in samples from the diseased sinus. However, the compositions of other microbiota did not show any statistically significant difference between the two sites (Supplementary Figure S3).

### 3.5. Relationship between Microbiota Abundance and LM CT Scores

**Figure 7:**
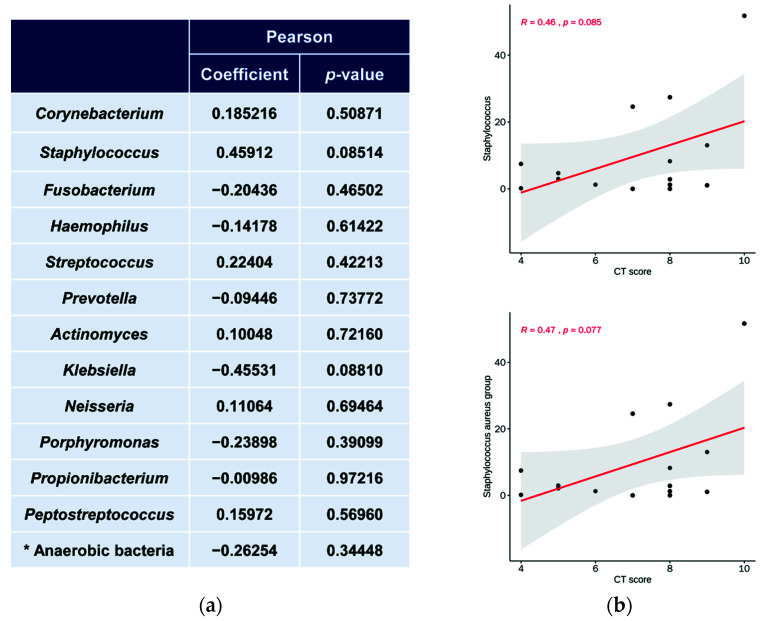
(**a**) Correlation between the relative abundance of microbiota at genus level and Lund–Mackay CT score at diseased site measured by Pearson correlation analysis. (**b**) Graphs of correlation of Staphylococcus (upper graph) and Staphylococcus aureus group (lower graph) with CT score. * anaerobic bacteria result includes Prevotella, Porphyromonas, Fusobacterium, and Propionibacterium. R, Pearson coefficient of correlation; CT score, Lund–Mackay CT score.

Finally, the relationship between clinical manifestation and microbiota composition was explored. Specifically, we examined the preoperative LM CT score corresponding to the diseased site (Table 1) and compared the same with the observed microbial composition. At the genus level, the relative abundance of *Staphylococcus*, one of the most important bacteria for microbial dysbiosis, was slightly correlated with the LM CT score (*p* = 0.08) (Figure 7a,b). Specifically, the abundance of *Staphylococcus aureus* at the species level tended to increase slightly with an increase in the LM CT score (Figure 7b). Other microbiota showed no significant correlation with the LM CT score at both the genus (Supplementary Figure S4) and species levels (Supplementary Figure S5).

## 4. Discussion

The crosstalk between innate immunity and microbiome composition is considered to occur via the microbiota integrating into the entire physiology of organisms and influencing multiple facets of organismal homeostasis via its effects on the innate immune system [9]. Further, microbiome contributes to epithelial barrier reinforcement, regulatory T cell induction, and also interacts with the innate immune system [46,47,48]. Even though the underlying mechanisms of CRS pathogenesis have not been clearly elucidated till date, hypothetically, it involves alterations in mucociliary clearance, abnormalities in epithelial barrier function, bacterial biofilms, and tissue remodeling in host innate and adaptive immune systems [49]. Additionally, in human upper airway, the sinonasal microbiome might play an important role in the pathogenesis of CRS, and based on advances in the culture-independent 16S rRNA sequencing technique, it has been proposed that dysbiosis is the initial cause of inflammation in CRS [50].

Many researchers have also used conventional bacterial culture methods for the identification of sinonasal bacteria, and have revealed that *Staphylococcus*, *Streptococcus*, and *Hemophilus influenza* are the commonly encountered microbiota in patients with CRS [21,51]; in this study, we also collected data from culture studies for reference (Table 1). However, culture-based studies are less informative compared with studies based on next-generation sequencing. Further, owing to the presence of non-culturable and unidentifiable microbiota, they are characterized by discrepancies in various sampling regions [21,52]. Specifically, advanced gene sequencing techniques have shed light on our clinical understanding regarding the effects of a diverse microbiome on human health [1,45].

In a recent study on inflammatory endotypes and microbial associations in CRS, bacterial community dysbiosis, including the depletion of *Corynebacterium*, *Propionibacterium*, and *Staphylococcus* spp., in patients with CRS, compared with the controls, has been reported [53].

Previous studies on the microbiome in patients with CRS have shown polymicrobial inflammation, suggesting the existence of complex interactions between the microbiome and the host. Further, in several studies, decreased diversity and richness in patients with CRS compared with the controls, have been reported [27,28,53,54,55]. Additionally, studies involving endoscopically guided brush samples of maxillary sinus revealed that the relative abundance of *Corynebacterium tuberculostearicum* increased, while that of *Lactobacillales, Carnobacterium alterfunditum, Enterococcus mundtii*, and *Pediococcus pentosaceus* decreased in patients with CRS, compared with the controls [53]. Studies involving bacteria and bacteria-derived extracellular vesicles from nasal lavage (NAL) fluid have also shown that patients with CRS showed a decrease in the proportion of *Bacteroidetes* and an increase in that of *Proteobacteria* at both phylum and genus levels. Further, the relative abundance of *Prevotella* showed a decrease, whereas that of *Staphylococcus*, belonging to the phylum *Firmicutes*, showed an increase in patients with CRS, compared with the controls [28]. Another study, involving swab samples from middle meatus, showed that *Corynebacterium* and *Staphylococcus* are dominant in both controls and patients with CRS, while *Streptococcus, Moraxella,* and *Haemophilus* showed lower relative abundances [27]. Additionally, comparing controls with patients with CRS suggested a more significant depletion in the relative abundance of typical health-associated bacterial taxa, including *Anaerococcus, Corynebacterium, Finegoldia, Peptoniphilus, Propionibacterium,* and *Staphylococcus* in the patients with CRS than in the controls [55].

However, increased bacterial diversity was observed in other studies [29,30]. Specifically, a study involving NAL fluid from middle meatus showed qualitatively similar microbiomes in patients with CRS and the controls; however, patients with CRS showed greater diversity and abundance of fungi. *Cryptococcus neoformans* was identified as the most abundant fungus in both groups, showing greater prevalence in patients with CRS [29].

Moreover, some studies had reported no significant differences in microbiota diversity between controls and patients with CRS [31,32]. Swab samples were obtained from the ethmoid sinus of 56 patients with CRS and 26 control subjects. Analysis showed a similarity in the biodiversity corresponding to the patients with CRS and the control groups at the phylum level. However, at the genus level, the relative abundances of *Propionibacterium* and *Porphyromonas* tended to decrease in the patients with CRS, more so than the controls. Interestingly, patients with better surgical outcomes had increased bacterial diversity at the time of surgery, along with a higher relative abundance of *Actinobacteria* [32].

In this study, we compared the sinonasal microbiome composition of unilateral diseased and contralateral healthy sites in patients with unilateral sinusitis. In each patient, the distributions of the local microbiome at the healthy and diseased sites were different, suggesting that local microbial dysbiosis possibly plays a significant role in the pathogenesis of CRS by altering the mucosal immune responses of sinonasal epithelium.

Further, it was also observed that at the genus level, *Corynebacterium* and *Staphylococcus* constituted the most common genera in both sinuses, with *Staphylococcus* showing a greater decrease in percentage relative abundance at the diseased sites than at the healthy sites. At the phylum level, *Actinobacteria* and *Firmicutes* showed a slight decrease, whereas *Proteobacteria, Bacteroidetes*, and *Fusobacteria* showed a slight increase at the diseased sites, more so than at the healthy sites. Notably, the anaerobe group showed a significant increase in relative abundance at the diseased site. This is in accordance with a previous report, indicating that anaerobes including *Fusobacteria*, *Parvimonas*, and *Prevotella* were found in unilateral maxillary CRS samples, but not in healthy samples [56].

*Staphylococcus* and *Corynebacterium* have previously been reported as the most common sinus microbiota [27,28,33,53]. *Staphylococcus aureus* colonizes the nares of 30% of people and other nasal microbiotas have strategies to interfere with its colonization [57]. The relative abundances of *Staphylococcus* spp. [27,28] and *Corynebacterium tuberculostearicum* [27,53] in the sinus mucosa of patients with CRS were determined in this study, and our results show that *Staphylococcus aureus* and *Corynebacterium tuberculostearicum* were significantly more decreased in samples from the diseased sites than in those from the healthy sites. These two species might be the “keystone microbiome species” that normally maintain a stable and interactive community, co-inhabiting with other microbiota in the healthy state [54]. A recently published study also showed that the *Corynebacterium* species were more abundant in the nasopharyngeal swabs of healthy controls compared with those of patients with otitis media [58].

Furthermore, based on our results, the diseased and healthy sites showed similar bacterial richness and diversity. The detailed mechanism of this bacterial diversity is still unclear and may be resolved by conducting additional studies in the future.

Moreover, another consideration is sinusitis of odontogenic origin, common cause of unilateral sinusitis. The difference in microbial findings has been reported between the patients with odontogenic sinusitis and the patients with rhinogenic chronic sinusitis with nasal polyp; predominance of anaerobic species in the odontogenic sinusitis [59,60]. Bacterial biofilms, assemblies of bacteria embedded in a self-produced polysaccharide matrix, also could be involved in the pathogenesis of odontogenic sinusitis [60].

In this study, it was also observed that the relative abundances of anaerobes, including *Prevotella*, *Porphyromonas*, *Fusobacterium*, and *Propionibacterium* in the samples from the diseased sites significantly increased. Reportedly, anaerobic genera are more abundant in the middle meatus of patients with CRS than in controls, and usually, sinus cavities are not anaerobic; therefore, augmentation of anaerobes might result from disease progression and pathology [61]. For example, *Fusobacterium,* which is associated with suppuration, can engender anaerobic conditions in paranasal cavities [62]. In several previous studies, the role of anaerobes in CRS has been investigated [56,63,64,65,66]. According to the reports, anaerobic organisms have been isolated from more than 50–100% of patients [66]. These results, along with the anaerobic microbiome dysbiosis observed in this study corresponding to samples from healthy and diseased sites, might demonstrate the role of anaerobes in CRS-related inflammation.

To investigate the association between clinical characteristics and microbial composition, we examined the LM CT scores corresponding to the samples obtained from diseased sites and their relationship with microbiota (Figure 7). Unexpectedly, the relative abundance of *Staphylococcus* tended to increase slightly with an increase in the LM CT score; this was not consistent with the genus-level microbial data, which showed a greater decrease in samples from diseased sites than in those from healthy sites (Figure 6). However, the examination of the relationship between the LM CT scores corresponding to samples obtained from the diseased sites, without comparison with those corresponding to the samples from the healthy sites, and the microbiota showed no statistically significant correlation. Thus, this study showed that LM CT score is not a critical association factor; this might need to be further verified by conducting further studies.

This study has some limitations, the first being the limited number of patients enrolled. Microbial composition from a larger population could add further weight to our data. Second, it would also be worthwhile to investigate whether the microbiota of a unilateral diseased site could be altered owing to surgery [37,38,39]. Third, further study is needed to examine the relationship between microbiota composition and another clinical manifestation such as nasal endoscopic findings (secretion or mucosa edema). Finally, the pathophysiological role of the microbiome in unilateral and bilateral CRS could differ; therefore, we are planning to perform further investigations in this regard by comparing our data on patients with unilateral CRS with those on patients with bilateral CRS.

## 5. Conclusions

In conclusion, we investigated the upper airway microbiome of patients with unilateral sinusitis. The microbiome of samples from unilateral diseased sites was compared with that of samples from contralateral non-diseased (healthy) sites, suggesting the importance of local microbial dysbiosis in CRS pathogenesis. We expect that these findings would contribute to further research on microbial dysbiosis in CRS and generate a deeper understanding of CRS pathogenesis.

## Figures and Tables

**Table 1:** Demographic and clinical characteristics of study population.

Subjects	Sex	Age	Underlying Diseases	Diseased Site	LM CT Score at Diseased Site	Blood Eo (%)	Allergy	Bacterial Culture at Lesion Side	Bacterial Culture at Normal Side
1	M	35	none	Rt	5	1.9	none	S. pneumoniae	no growth
2	F	34	DM, Pul.Tb	Rt	8	0.9	none	S. aureus, S. epidermidis	no growth
3	M	54	DM	Lt	7	2.2	D. farinae, house dust	P. aeruginosa, G (-) bacilli	no growth
4	M	32	none	Rt	4	1.7	none	E. aerogenes	no growth
5	F	17	none	Rt	9	0.11	none	H. parainfluenzae	E. aerogenes
6	M	48	none	Rt	6	3.5	none	S. aureus (MRSA)	S. aureus
7	M	56	none	Lt	4	4.2	none	S. epidermidis	S. epidermidis
8	M	67	HTN, Hepatitis B	Lt	8	2.3	Cladosporium	S. aureus, G (+) cocci	S. aureus, G (+) cocci, G (−) bacilli
9	M	59	none	Rt	9	5.2	none	S. epidermidis, G (+) bacilli, Corynebacterium Species	S. epidermidis
10	M	34	none	Rt	10	2.3	D. farinae, D. pteronyssinus	S. aureus	S. aureus
11	M	54	none	Lt	8	1.8	D. farinae, house dust, storage mite, Acarus siro, cockroach, Cladosporium, Aspergillus	E. aerogenes	S. epidermidis
12	M	57	none	Lt	7	4.4	D. farinae, D. pteronyssinus, house dust, storage mite, cockroach, multiple tree, grass, weed pollens	S. anginosus	S. aureus
13	M	39	none	Lt	5	4.8	none	S. epidermidis, S. aureus	no growth
14	M	42	hyperthyroidism	Rt	8	0.12	D. farinae, D. pteronyssinus, house dust, Acarus siro	E. aerogenes	no growth
15	M	65	DM, HTN	Rt	8	0.5	none	K. aerogenes	K. aerogenes

LM CT score, Lund–Mackay CT score; Blood Eo, blood eosinophils; DM, diabetes mellitus; HTN, hypertension; Pul.Tb, pulmonary tuberculosis; D. farinae, Dermatophagoides farinae; D. pteronyssinus, Dermatophagoides pteronyssimus; S. pneumoniae, Streptococcus pneumoniae; S. aureus, Staphylococcus aureus; S. epidermidis, Staphylococcus epidermidis; P. aeruginosa, Pseudomonas aeruginosa; E. aerogenes, Enterobacter aerogenes; MRSA, methicillin-resistant Staphylococcus aureus; H. parainfluenzae, Haemophilus parainfluenzae; S. anginosus, Streptococcus anginosus; K. aerogenes, Klebsiella aerogenes.

## Data Availability

The data used to support the findings of this study are available from the corresponding author upon request.

## Supplementary Materials

The following are available online at https://www.mdpi.com/article/10.3390/ijerph18189878/s1, Figure S1: Comparison of bacterial species richness and alpha diversity in samples of healthy site and diseased site. Richness represented by (a) Jackknife estimation. Alpha diversity examined by (b) Simpson index, and (c) phylogenetic diversity. Beta diversity examined by (d) principal coordinates analysis (PCoA) plots, Figure S2: Comparison of microbiota between healthy site (upper bar) and diseased site (lower bar) at species level. Stacked bars show average taxonomic composition of selected communities of bacterial species level, Figure S3: Composition of representative microbiota at species level. Paired line plots show relative abundance of selected communities in comparison between healthy site and diseased site, Figure S4: Graphs of correlation between representative microbiota at genus level and Lund–Mackay CT score at diseased site, Figure S5: Graphs of correlation between representative microbiota at species level and Lund–Mackay CT score at diseased site, Table S1: Results from sequence read counts.
