# Supplementary material for: Microbiome of Unilateral Chronic Rhinosinusitis: A Controlled Paired Analysis
Source: Int J Environ Res Public Health. 2021 Sep 19;18(18):9878. doi: 10.3390/ijerph18189878 (PMC8469123; doi:10.3390/ijerph18189878)
Supplement: Supplementary file 1 [file ijerph-18-09878-s001.zip › ijerph-1354583-SI.pdf]

Supplementary Materials

SUPPLEMENTARY FIGURES

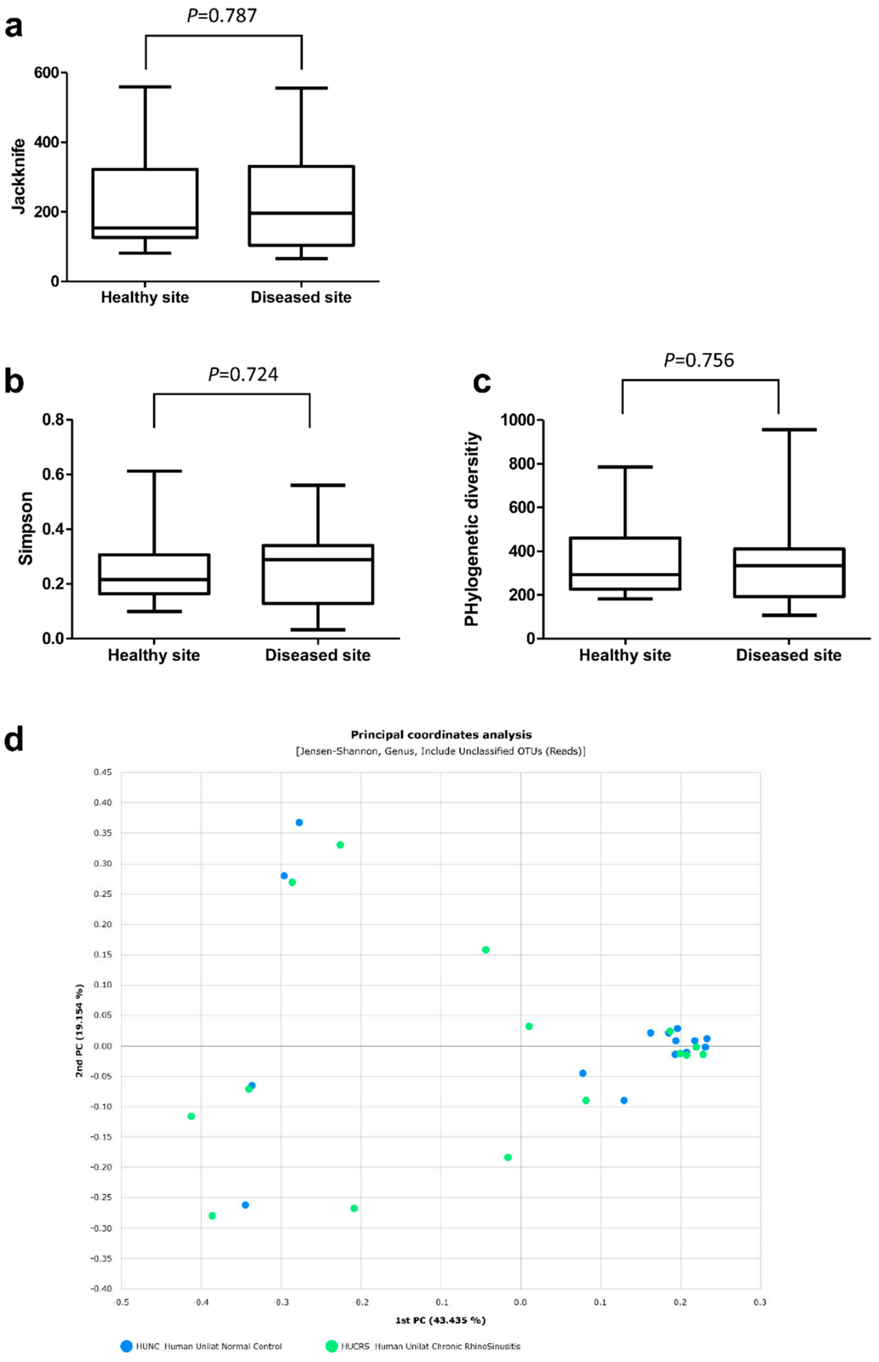

Supplementary Figure S1. Comparison of bacterial species richness and alpha diversity in samples of healthy site and diseased site. Richness represented by (a) Jackknife estimation. Alpha diversity examined by (b) Simpson index, and (c) phylogenetic diversity. Beta diversity examined by (d) principal coordinates analysis (PCoA) plots.

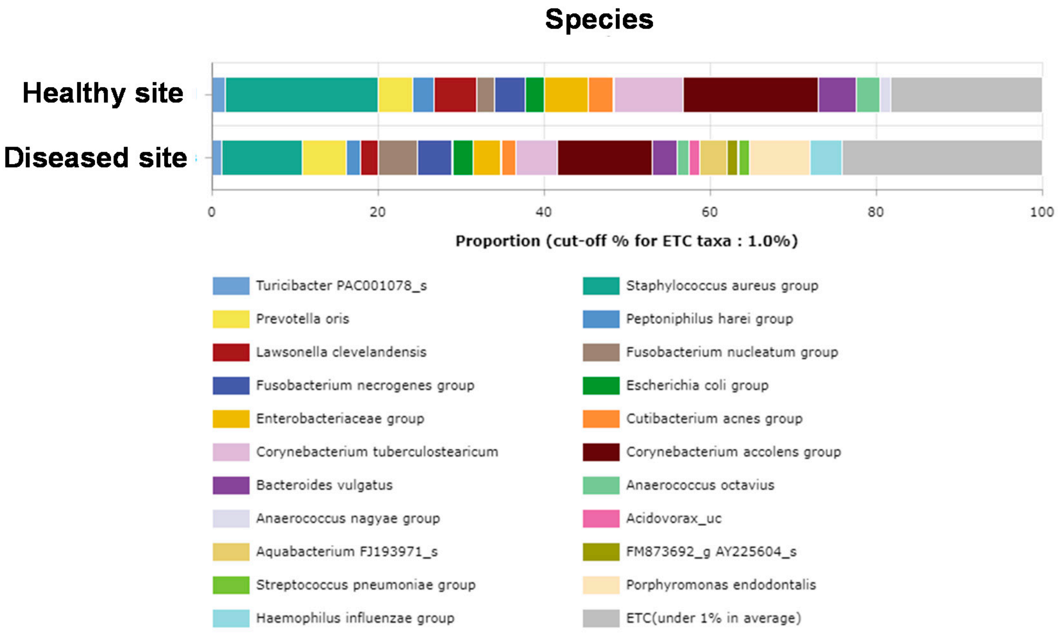

Supplementary Figure S2. Comparison of microbiota between healthy site (upper bar) and diseased site (lower bar) in species level. Stacked bars show average taxonomic composition of selected communities of bacterial species level.

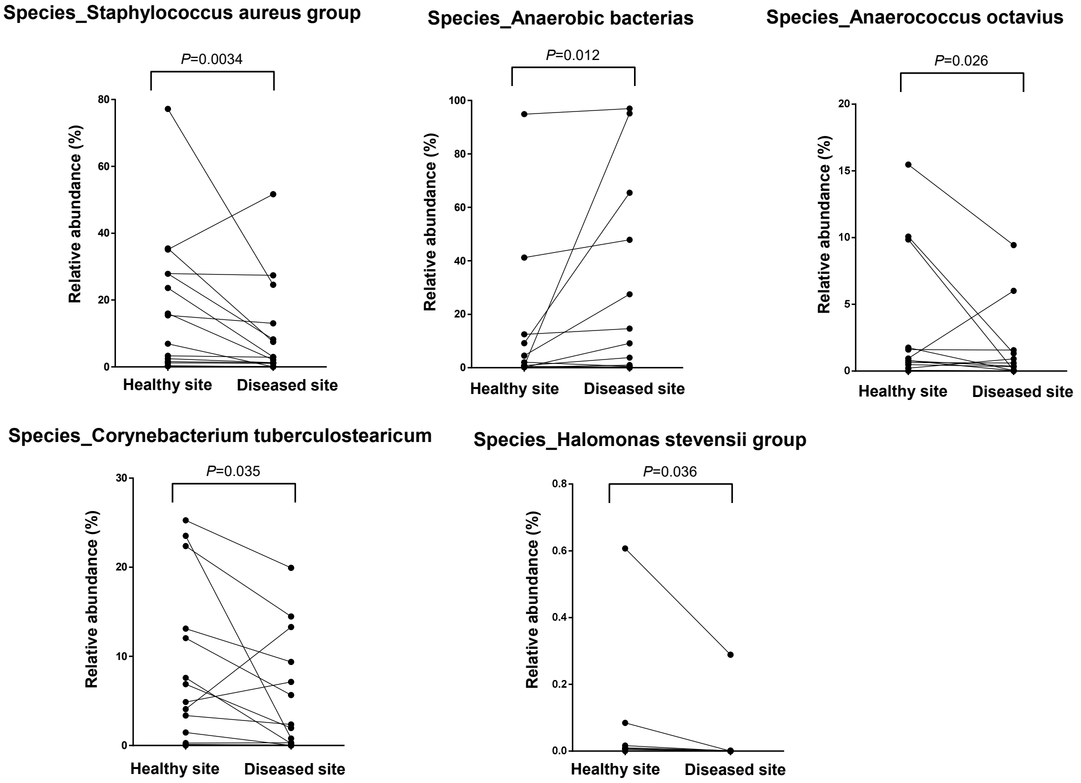

Supplementary Figure S3. Composition of representative microbiota at species level. Paired line plots show relative abundance of selected communities in comparison between healthy site and diseased site.

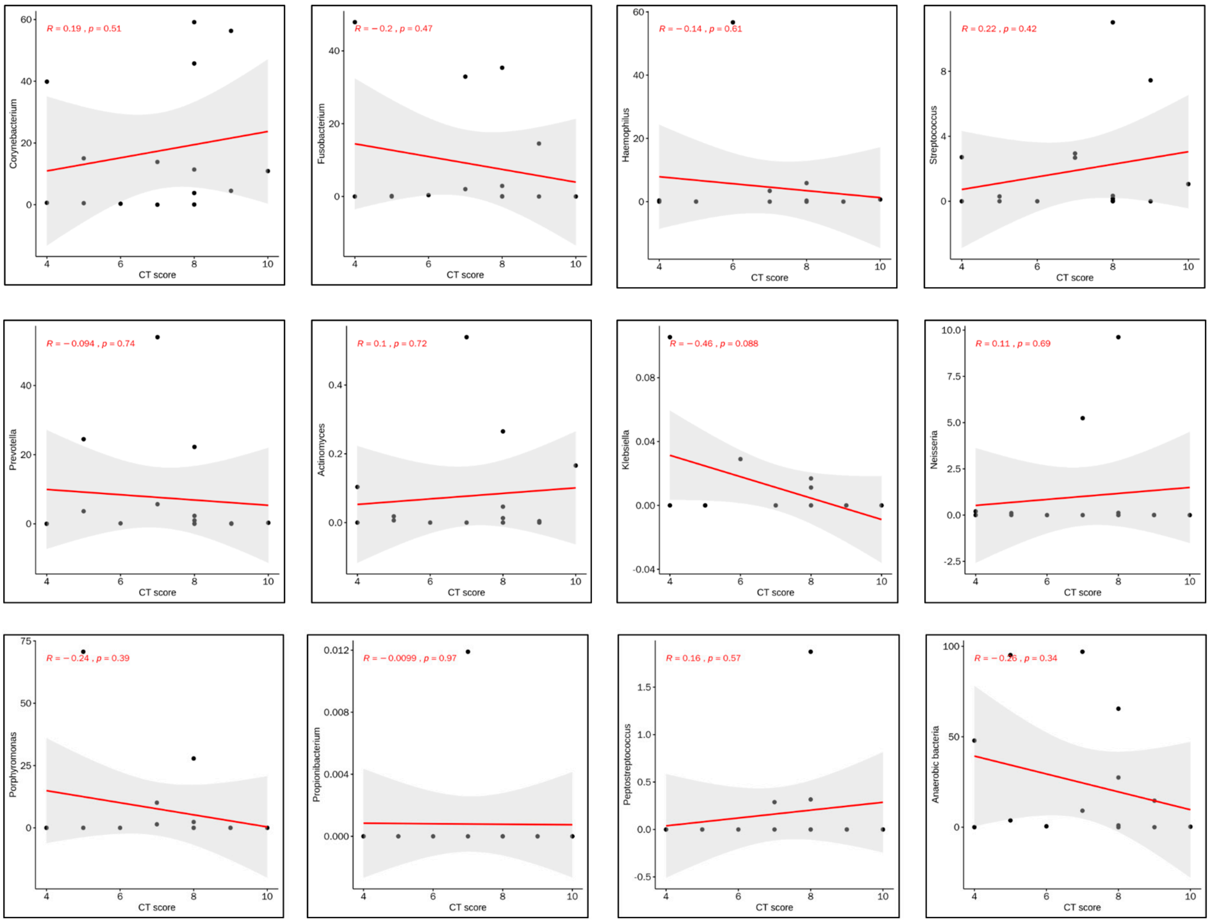

Supplementary Figure S4. Graphs of correlation between representative microbiota at genus level and Lund-Mackay CT score at diseased site.

R, Pearson coefficient of correlation; CT score, Lund-Mackay CT score.

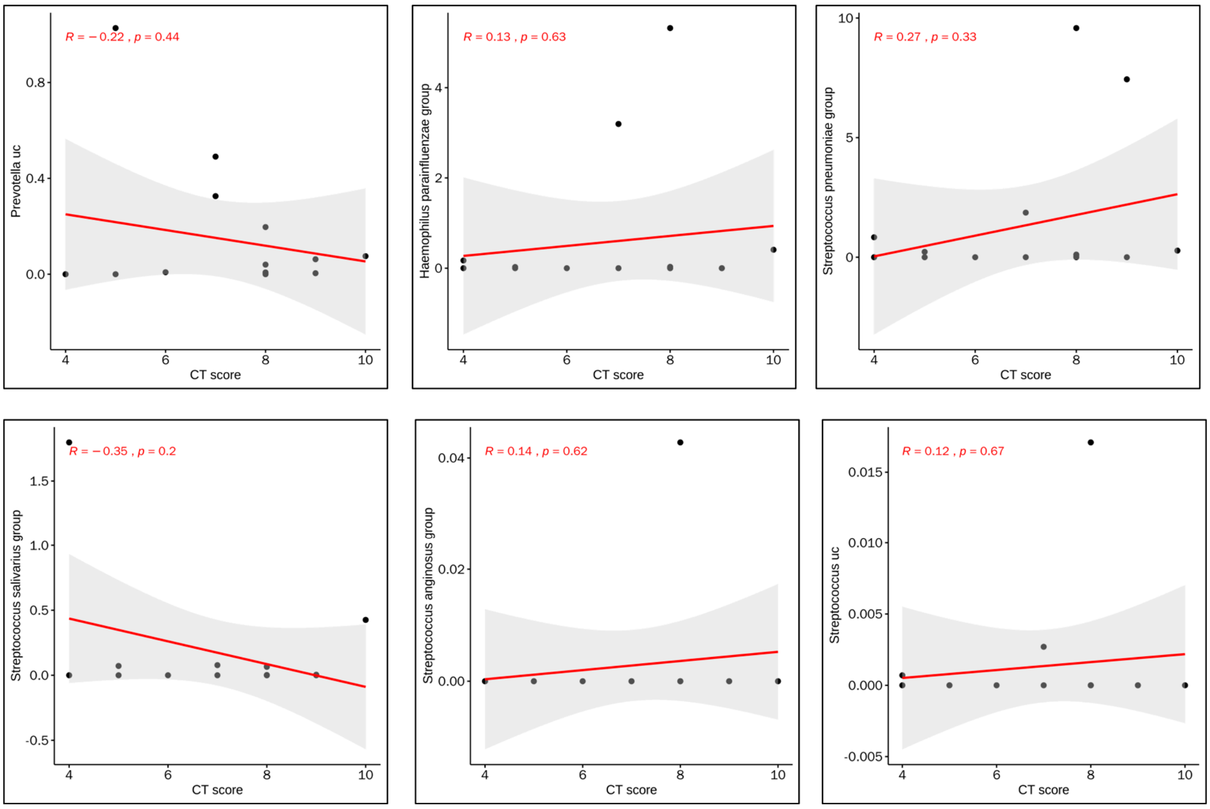

Supplementary Figure S5. Graphs of correlation between representative microbiota at species level and Lund-Mackay CT score at diseased site.

R, Pearson coefficient of correlation; CT score, Lund-Mackay CT score.

Supplementary Table S1. Results from sequence read counts

Subjects	Total reads after pre-filter	Total valid reads	Reason of removed			Length			Identified at the species level	%	No of species
			Low quality amplicons	Non-target ammplicons	Chimeric amplicaons	min	average	max			
**Healthy site**											
1	54717	42920	11629	5	163	399	414.7	445	42369	98.7	66
2	209550	201340	7579	3	628	384	414.7	448	200381	99.5	120
3	103114	85157	17641	6	310	148	425	454	84812	99.6	83
4	136336	104353	9354	19517	3112	346	417.1	449	103561	99.2	81
5	38839	28028	1173	27	9611	395	413.4	445	26709	95.3	352
6	33591	23856	1026	2	8707	398	415.6	445	22893	96	258
7	65937	56829	1602	2	7504	237	417.3	446	55089	96.8	237
8	49593	46109	1539	1	1944	397	413.9	449	45791	99.3	74
9	53802	51049	2122	11	620	260	413.6	448	49997	97.9	67
10	65060	60991	3211	32	826	304	413	449	60844	99.8	123
11	68461	65097	2185	22	1157	153	409.3	444	64822	99.6	73
12	51147	39300	11493	2	352	400	417.2	455	39110	99.5	111
13	220625	209573	9855	254	943	160	413.1	448	208560	99.5	181
14	23153	19067	3903	80	103	237	414.4	448	18976	99.5	226
15	50670	10613	39894	0	163	237	411.2	447	10420	98.2	105
**Diseased site**											
1	104645	92122	5608	1356	5559	256	412.2	448	86304	93.7	443
2	245466	236900	8088	4	474	260	424.6	448	191401	80.8	91
3	107821	92540	14801	2	478	148	421.3	449	91081	98.4	213
4	159721	136016	10726	11778	1201	394	413.6	454	134929	99.2	82
5	32645	24009	1090	0	7546	398	4140	440	23140	96.4	256
6	29205	24108	773	0	4324	394	421.6	445	23602	97.9	177
7	69966	61691	1633	0	6642	256	416.1	446	60148	97.5	186
8	56200	53322	1762	31	1085	384	414	449	52844	99.1	153
9	50320	47741	1639	18	922	260	412	447	47294	99.1	55
10	64128	58460	5491	49	128	260	417	449	58256	99.7	90
11	54784	51255	1927	18	1584	397	414	462	50863	99.2	49
12	92199	73349	16109	1	2740	260	416.6	456	73105	99.7	29
13	304343	289235	13679	58	1371	397	422.6	445	289190	100	63
14	46283	44677	1445	0	161	399	412.4	444	44527	99.7	140
15	30855	11691	18969	0	195	259	418.5	448	11606	99.3	166
